# Is the Conjunctiva a Potential Target for Advanced Therapy Medicinal Products?

**DOI:** 10.3390/pharmaceutics13081140

**Published:** 2021-07-26

**Authors:** Yolanda Diebold, Laura García-Posadas

**Affiliations:** 1 Ocular Surface Group, Instituto de Oftalmobiología Aplicada (IOBA), Universidad de Valladolid, 47011 Valladolid, Spain; lgarciap@ioba.med.uva.es; 2 Centro de Investigación Biomédica en Red de Bioingeniería, Biomateriales y Nanomedicina (CIBER-BBN), Instituto de Salud Carlos III, 28029 Madrid, Spain

**Keywords:** advanced therapies, cell therapy, conjunctiva, ocular mucosa, gene therapy, tissue engineering

## Abstract

The conjunctiva is a complex ocular tissue that provides mechanical, sensory, and immune protection for the ocular surface. It is affected by many diseases through different pathological mechanisms. If a disease is not treated and conjunctival function is not fully restored, the whole ocular surface and, therefore, sight is at risk. Different therapeutic approaches have been proposed, but there are still unsolved conjunctival alterations that require more sophisticated therapeutic options. Advanced therapy medicinal products (ATMPs) comprise a wide range of products that includes cell therapy, tissue engineering, and gene therapy. To the best of our knowledge, there is no commercialized ATMP specifically for conjunctival treatment yet. However, the conjunctiva can be a potential target for ATMPs for different reasons. In this review, we provide an overview of the advances in experimental phases of potential ATMPs that primarily target the conjunctiva. Important advances have been achieved through the techniques of cell therapy and tissue engineering, whereas the use of gene therapy in the conjunctiva is still marginal. Undoubtedly, future research in this field will lead to achieving commercially available ATMPs for the conjunctiva, which may provide better treatments for patients.

## 1. Introduction

The human conjunctiva is a complex and fascinating ocular tissue. Traditionally neglected in favor of the cornea, its functions are essential in maintaining ocular surface homeostasis. Over the years, as our knowledge of tear film complexity and the pathophysiology of the ocular surface has increased, the conjunctiva has slowly come to be acknowledged as an essential protective element for ocular surface structures. MUC5AC, a well-known mucin specifically secreted by the conjunctival goblet cells, as well as many other different secretory products, participates in the maintenance of the tear film [1,2].

The conjunctiva is a mucosal tissue that extends from the mucocutaneous junction at the lid margin to the limbal region next to the peripheral cornea and rests on the sclera. In essence, the role of the conjunctiva is to protect the transparency of the cornea, a much more vulnerable tissue that lacks blood and lymphatic vessels, as well as a sufficiently strong in situ immune response for full protection from foreign invaders.

Due to its anatomical features and many roles, the conjunctiva is difficult to study and model in the laboratory. We now know that conjunctival pathophysiology is complex and affects the homeostasis of the so-called lacrimal functional unit [3,4]. Conventional pharmacological treatments are not sufficient or curative in many instances for recovering a functional conjunctiva and/or maintaining healthy ocular surface tissues.

The aim of this review is to present the challenges that the conjunctiva poses from clinical and therapeutic points of view and analyze the reported developments that may become advanced therapies for conjunctival diseases in the future.

### 1.1. Conjunctival Structure and Functions

From an anatomical point of view, the conjunctiva is generally divisible into three main regions: (1) the tarsal, or palpebral, which lines the inner surface of the eyelids; (2) the forniceal, which lines the upper and lower fornices; and (3) the bulbar, which overlays the sclera on the anterior portion of the globe. These three regions are specialized in different functions, ranging from trapping small foreign objects in a net of secreted mucins and facilitating their removal by blinking to providing immune protection to the cornea by the local presence of lymphoid tissue [5].

The complexity of the conjunctiva relies upon the multiple tissues present in its structure: (1) a non-keratinized stratified squamous epithelium that possesses five reported epithelial cell subtypes, including goblet cells; (2) a basal membrane, where potential autoantigens reside and, subsequently, immune material becomes deposited in certain autoimmune diseases (e.g., mucous membrane pemphigoid), in addition to a loose stroma mainly composed of type IV collagen; (3) an abundant vasculature; (4) a regional lymphoid tissue—namely, conjunctiva-associated lymphoid tissue (CALT) and lymphatic vessels; (5) a melanocyte population; and (6) sensory afferent nerve fibers derived from the ophthalmic (in the bulbar and palpebral areas) and the maxillary (in the inferior forniceal area) branches of the trigeminal nerve. In addition, the conjunctiva possesses the accessory lacrimal glands of Wolfring and Kruse, which are present in the tarsal conjunctiva and in the fornices, respectively, and the pseudoglands of Henle, which are groups of abundant goblet cells that also appear in the tarsal plate.

**Figure 1:**
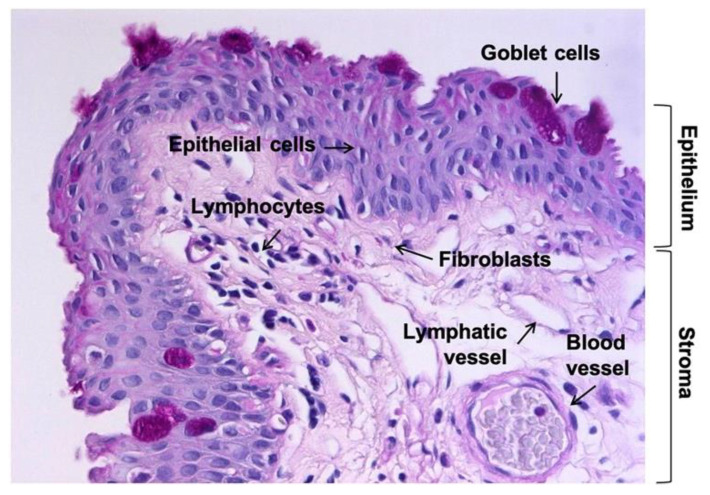
Tissue section of human conjunctiva with periodic acid-Schiff (PAS) staining showing the main cell types present in the epithelium and stroma. Magnification: 200×.

An amazing variety of cell types are part of the conjunctiva (Figure 1) and account for its functional complexity: mucin-secreting epithelial cells, fibroblasts, melanocytes, dendritic cells, lymphocytes, eosinophils, neutrophils, and mast cells, not to mention mesenchymal stem cells (MSCs). This illustrates how difficult it has been to model conjunctival tissue in the laboratory over the years.

From a functional point of view, the conjunctiva realizes the mechanical, sensory, and immune protection of the ocular surface; the specialized secretion of fluid, electrolytes, and diverse components of the tear film [2], mainly mucins and antimicrobial peptides [6]; the modulation of the local inflammatory state; the regulation of tissue repair and fibrosis; neo-angiogenesis; and pain perception [7]. In addition, epithelial cells can respond to signals derived from the abundant microbiota resident on the ocular surface producing inflammatory cytokines [8]. Further, it is worth mentioning the contribution of the conjunctiva to the antioxidant system protecting the ocular surface with the expression of superoxide dismutase, catalase, glutathione synthetase, and glutathione reductase [9] and peroxiredoxin I [10], in addition to glutathione [11].

This wide range of functions makes the conjunctiva a key element in the maintenance of ocular surface homeostasis and, at the same time, quite reactive to small environmental changes and even prone to alterations. This aspect will be further discussed in this review.

### 1.2. Regeneration of the Conjunctival Tissue

Tissue regeneration allows the complete functional recovery of damaged tissue, while tissue repair usually leaves structural alterations or even permanent scars associated with collagen deposits, which may lead to disorders. The regeneration process relies upon the local presence of stem cells, whose activation and proliferation lead to the replenishment of lost cells. Ocular surface epithelia are able to self-renew; however, conjunctival regeneration is still debated among experts. The main reasons for this are related to the lack of clarity regarding the presence of human conjunctival stem cells in the different conjunctival regions and their potential to regenerate not only squamous epithelial cells but also conjunctival goblet cells, as discussed below.

There are few published papers for which the potential locations of epithelial stem cells in the human conjunctiva have been studied. The currently accepted locations are the fornix [12,13] and the bulbar conjunctiva [12,13,14,15,16]. In one of the latest papers on this topic published to date, clonogenic ability and stem cell marker expression in both fixed tissue and cultured cells from the same human donors were used to identify conjunctival stem cells, resulting in stem cells being scattered throughout the basal epithelial cell layer of the whole conjunctival tissue. However, the highest levels of stem cell markers are located in the medial canthal and inferior forniceal areas of the conjunctiva [17] with no apparent organization in a niche, as is the case in the limbus. We still know very little about the mechanism of conjunctival epithelial renewal and how the bipotent cell precursor proposed by Pellegrini et al. in 1999 [14] actually gives rise to either a squamous cell or a goblet cell. What is clear, however, is the fact that conjunctival tissue may fail to regenerate and give rise to pathology, as limbal and corneal tissues do [18,19].

Additionally, the conjunctival stroma possesses multipotent MSCs that express markers of undifferentiated stem cells [20]. Several studies have demonstrated the capacity of these conjunctival MSCs to differentiate into corneal cells [21], photoreceptor cells [22], or insulin-producing cells [23,24], among others, suggesting their utility for tissue engineering and ocular reconstruction [22,25].

### 1.3. In Vitro/Ex Vivo Systems for Studying Conjunctival Pathophysiology

As mentioned previously, the conjunctiva has long been neglected. The lack of research in this field means that there are few models with which to study the tissue, and fewer models mean less knowledge. This vicious circle needed to be broken, and fortunately, it seems that it actually has been. Although the in vitro models available with which to study the normal functioning of the conjunctiva and the diseases affecting it are limited in number, they have been improved over the last few years. Several new immortalized cells and more complex cell culture models have been added to the “classic” cell lines. The complexity of these models ranges from cell monolayers of a single cell type to complete 3D models that can more faithfully represent the structure of this tissue [26].

The main advantage of cell lines is that they are easy to use and allow the obtaining of large quantities of cells with which to perform many experiments. However, they can show important differences from the native tissue cells [27]. The spontaneously immortalized Wong–Kilbourne derivative of the Chang cell line has been widely used in conjunctival research. However, it lacks the expression of typical markers such as cytokeratin (CK) 4 and the adhesion protein E-cadherin. It also differs from normal primary cultures of the human conjunctiva in its response to inflammatory cytokines [28], and, in addition, it is commonly acknowledged that it is cross-contaminated with HeLa cells [29,30]. For that reason, it is not frequently used today. Another spontaneously immortalized cell line is IOBA-NHC, which was developed by Diebold et al. [31]. It has allowed us to increase our knowledge of the inflammatory response of conjunctival epithelial cells [32,33,34], but, unfortunately, it has shown signs of senescence, limiting its use in the last few years. Another widely used cell line is telomerase-immortalized human conjunctival epithelial cells (ConjEp-1/p53DD/cdk4R/TERT, abbreviated to HCjE), which were developed by Gipson et al. from primary cultures [35]. HCjE cells express some of the markers typically found in the native conjunctival epithelium, such as CK19 and MUC1, 4 and 16. However, the expression of MUC5AC is sparse. Finally, another immortalized human conjunctival epithelial cell line (IM-HConjEpiC) has been commercialized by Innoprot, Innovative Technologies in Biological Systems, S.L. (Derio, Spain). IM-HConjEpiCs were developed by immortalizing primary human conjunctival epithelial cells with SV40 large T antigens. Although these cells may be a valuable tool, further phenotypic and functional characterization is needed.

Other than the existence of cell lines representing normal conjunctiva, some authors have established cell lines for different diseases, such as conjunctival squamous cell carcinoma [36].

To overcome some of the limitations of cell lines, several authors have described different protocols for isolating and culturing primary cells from the human conjunctival epithelium [37,38,39] and stroma [39]. Conjunctival goblet cells have also been cultured from rat [40], human [41], and mouse [42] tissues. Finally, to study diseases affecting the conjunctiva, cells can be directly isolated from pathological tissue. These cells can be expanded in vitro and used to study the physiopathology of the pterygium [43,44,45] or the ocular pemphigoid [46], among others.

All these cell culture systems have allowed researchers to analyze the response of the conjunctiva to inflammatory stimuli, perform the initial screening of different drugs, or study the signaling pathways involved in mucin secretion [47,48,49,50]. However, they are limited in their capacity to represent the complex connections between the different cell types that compose the conjunctiva. This can only be partially achieved with the use of more complex 3D models. We recently reviewed the available human 3D cell culture models of the anterior segment of the eye, including the conjunctiva [26]. Some of these models only represent the epithelium [51], and some others also include a fibroblast-containing stroma mimicked by a scaffold made from collagen [52] or fibrin [53]. There is no doubt that more complex and representative 3D models of the conjunctiva will be constructed with the aid of tissue engineering in the near future.

Finally, there are several ex vivo models of the conjunctiva. Tovell et al. described an ex vivo model used to study conjunctival scarring [54]. They maintained ex vivo segments of porcine conjunctiva in culture and analyzed the tissue contraction in response to different substances. Although this model uses the porcine conjunctiva, it could probably be adapted to human tissue.

## 2. Diseases Affecting the Conjunctiva

The conjunctiva is involved in a wide variety of ocular surface disorders, in which it becomes damaged to different extents. The mechanisms that lead to the development of alterations in conjunctival tissues include infectious, autoimmune and immune-based, cicatrizing, and inflammatory diseases; benign and malignant tumors; and chemical trauma. In some of these conditions, a wide area of the diseased conjunctiva must be removed, and the subsequent wound has to be covered with another tissue. When a conjunctival wound is neglected, serious damage develops; a secondary healing of the conjunctiva occurs and leads to dysfunctional conjunctival scarring. In turn, conjunctival scarring reduces the motility of the eyeball, which can result in severe anatomical and functional impairment, such as the development of diplopia. In addition, there may be a loss of the conjunctival secretory cells, such as goblet cells and accessory lacrimal glands of Wolfring and Kruse, which alters the conjunctiva’s contribution to a healthy tear film and leads to additional damage to the ocular surface. This scenario clearly shows how relevant it is to achieve complete functional regeneration of the conjunctival tissues.

Table 1 summarizes the main diseases that can affect conjunctival tissues and may require tissue transplantation in a way that is intended to be informative rather than exhaustive. The ideal conjunctival tissue graft would be healthy conjunctival tissue from the same or the contralateral eye. However, an autologous healthy conjunctiva is not always available, especially in recurrent and/or bilateral cases.

The current conventional regenerative treatments for the conjunctiva mainly involve non-ocular tissues, such as the amniotic membrane (AM) and oral/nasal mucosa. The AM is the innermost placental layer and possesses a multilayered structure in which mesenchymal stem cells are present. The main biological properties of the AM include a lack of immunogenicity, as well as anti-fibrotic, anti-inflammatory, anti-angiogenic, and antimicrobial features. The AM can be used as a basement membrane substitute or as a temporary graft. Its use in ocular surface reconstruction has expanded since 1995, mainly because of its transparency and ability to promote epithelialization [55]. When grafted in conjunctival defects, AM supports conjunctival re-epithelialization when conjunctival stem cells remain in the recipient and helps to repopulate the tissue [56].

There are many published examples regarding the use of oral or nasal mucosal tissues for reconstructing conjunctival defects. For a comparative review, see [57]. Oral mucosal grafting remains the most viable option for the replacement of the conjunctiva in the absence of autologous healthy tissue; however, its main limitation is the lack of goblet cells, along with cosmetic issues. Nasal mucosal grafts maintain goblet cells, and, for some indications, they may be preferred to oral mucosal transplants [58].

In many instances, AM is not sufficiently effective; in the most frequent indication—after pterygium removal surgery—a conjunctival autograft is more effective than AM [59], but the conjunctiva is not always available, as mentioned before. As another example, when the entire conjunctival fornices and palpebral conjunctiva need to be reconstructed, the oral or buccal mucosa is used if there is no other option. However, the results are not functionally or esthetically optimal, and, in many of the indications, the oral mucosa is also compromised with the same background disease; in this case, if the eyelid mucosa is not satisfactorily reconstructed, all attempts to restore vision by corneal transplant are doomed to fail [60].

Therefore, it is clear that there is a clinical need for human healthy conjunctival tissue that regular tissue sources cannot satisfy. Considering this fact, the development of advanced therapy medicinal products (ATMPs) may have enormous potential to help in conjunctival functional regeneration.

Other than the need to cover an extensive conjunctiva tissue area after the removal of diseased tissue, there is a common biological situation in most of the conjunctival pathologies included in Table 1: The presence of fibrosis.

Fibrosis is a complex biological process that is related to different diseases that potentially cause blindness. Fibrotic diseases are characterized by tissue contraction as a result of fibroblast activation and the excess accumulation of the extracellular matrix. Different cells can be involved in the process; however, myofibroblasts, which are activated fibroblasts, play a pivotal role. Scarring is an aberrant wound healing process that results in the formation of a permanent scar that affects not only the tissue morphology but also the functional recovery of wounded tissues. The cytokine transforming growth factor-beta (TGFβ) is the main fibrogenic signal that modulates the fibrotic process [61,62].

The conjunctiva, along with the cornea, is susceptible to fibrotic disease [63]. We can find fibrovascular scarring underlying different situations, such as pterygium, ocular pemphigoids, Stevens–Johnson syndrome, ocular graft versus host disease, or glaucoma filtering surgery (trabeculectomy) [64]. In these, there is an underlying inflammatory or wound healing alteration that triggers the formation of fibrotic tissue. For instance, it is well-established that TGFβ mediates scarring in the conjunctiva, which, in turn, can lead to a reduction in filtration efficacy after trabeculectomy [65]. Another example is pterygium, a very common multifactorial disorder of the conjunctiva that includes an ingrowth of fibrovascular subconjunctival connective tissue, among other features [66,67]. Currently acknowledged as a proliferative disorder more than a degeneration of the conjunctival stroma, pterygium involves a cicatricial fibrotic alteration that can eventually become very severe and impair globe motility and even vision.

For those reasons, the development of effective anti-scarring therapies could represent a revolution in the management of ocular surface diseases and tissue injuries, including surgery.

## 3. Potential of Advanced Therapy Medicinal Products (ATMPs) to Improve Conjunctival Treatment

The conjunctival diseases listed in Table 1, in addition to their specific pharmacological therapies, may be candidates for advanced therapies.

To the best of our knowledge, there is no commercialized ATMP specifically for conjunctival treatment yet. There are several developments that have the potential to become ATMPs in the near future, as explained below. In general, the term ATMPs groups somatic cell therapy medicinal products, tissue engineering products, gene therapy medicinal products, and the combination of any of the previous with a medical device (combined ATMP) [68].

### 3.1. Cell-Based Therapies

Tissue engineering and cell therapy are emerging disciplines that combine biomaterials, bioengineering, and cell biology to repair or regenerate biological tissues [69]. Tissue engineering involves developing polymeric scaffolds and assembling them together with cells and/or biologically active molecules to construct bioengineered tissues with features similar to those of the original tissue so that they are able to renew, regenerate, or replace damaged tissues [70,71]. Different cell types, including stem cells, can be expanded ex vivo and stimulated in different ways to achieve the differentiation of several cell types or allow better performance to be obtained.

Regenerative medicines for eye tissues focused on tissue engineering techniques have been developed and established as a new clinical field with enormous potential. In particular, the regeneration of ocular surface tissues such as the cornea or the limbus has greatly benefited from diverse tissue engineering developments (for a recent review, see [72,73]). Regarding human conjunctival tissue regeneration, some examples have been described in preclinical studies, but most of them have not yet been investigated in clinical studies. However, it is clear that there is a clinical need for healthy human conjunctival tissue that regular tissue sources cannot satisfy. Bioengineered tissues are considered an appealing solution for use as ATMPs for severe ocular surface disorders involving the conjunctiva. Additionally, the in vitro recapitulation of conjunctival tissues for transplantation seems to be a promising strategy along with their ex vivo expansion [74]. Two clinical studies have analyzed the efficiency of using human conjunctival tissue expanded ex vivo to regenerate the ocular surface [75,76].

Ricardo et al. expanded a forniceal conjunctiva biopsy on the basement membrane surface of denuded AM [75]. After two weeks in culture, conjunctival epithelial cells were transplanted on the corneal surfaces of 12 eyes from 10 patients with chemical burns, idiopathic ocular surface disease, or Stevens–Johnson syndrome, among other conditions. After the transplantation, the authors observed re-epithelialization with the transparent and regular epithelium, achieving partial or total success in 10 out of 12 eyes. This study demonstrates the capacity of cultured conjunctival epithelial cells to restore the ocular surface.

In 2014, Vasania et al. published the results of a multicentric clinical trial performed in India with the purpose of establishing “the efficacy and safety of ex vivo cultured autologous human conjunctival epithelial cell transplantation for treatment of pterygia” [76]. Similar to the procedure described by Ricardo et al., they obtained superior fornix biopsies and seeded them on AM. Cells were cultured for 14–21 days before using them as grafts to cover the conjunctival defect performed during pterygium surgery. No significant complications were reported, and the pterygium recurrence rate was 21.7%. Interestingly, 82.6% of the patients showed adequate goblet cells present at the site of transplantation.

Di Girolamo et al. developed a method to expand and transplant autologous conjunctival stem cells onto the ocular surface by using contact lenses as carriers [77,78]. A biopsy was obtained from the superior forniceal conjunctiva, placed on the concave surface of a siloxane-hydrogel contact lens, and cultured until the cells reached confluence. Then, the contact lens was inserted into the patient’s eye. With this technique, the authors achieved a successful reconstitution of the ocular surface using autologous cells even in cases of bilateral disease. Interestingly, more successful outcomes were obtained with conjunctival cells (78%) than with limbal cells (43%) [78]. Another advantage of this method is that transplanted cells are not exposed to foreign human biological or xenogeneic materials.

These examples highlight the potential of using ex vivo expanded conjunctival epithelial cells to successfully treat different pathological conditions affecting the ocular surface. Nevertheless, there are other examples of using non-ocular engineered cells that have achieved promising results. Kobayashi et al. engineered ex vivo-expanded nasal mucosal epithelial cells from biopsy-derived human nasal mucosal tissues on AM [79]. Interestingly, the bioengineered tissue was stratified and included a high density of functional goblet cells. When transplanted onto defective conjunctival areas surgically created in rabbits, the generated tissues survived and remained clear and smooth two weeks after transplantation, without signs of extensive inflammation. The expression of several markers, including MUC5AC mucin, was detected in the transplanted tissue. Although this study did not elucidate the molecular pathway involved in the differentiation of transplanted nasal tissue, the results were quite promising, as it is difficult to maintain functional goblet cells in culture. 

An early attempt was published by Yang et al. in 2015, in which the feasibility of the directed differentiation of human amniotic epithelial cells into the conjunctival epithelium was tested [80]. The transformation of conjunctival epithelial cells after AM transplantation to repair conjunctival damage due to burns had previously been reported [81,82]. Amniotic epithelial cells at passage 3 were used to inoculate a human decellularized conjunctival matrix and left for five days to differentiate. They differentiated into cells with the phenotype of conjunctival goblet and non-goblet epithelial cells expressing markers such as cytokeratin (CK 4, CK 13) and the goblet cell-associated mucin MUC5AC. Then, the authors constructed an engineered conjunctiva using a decellularized amniotic membrane as a scaffold and amniotic epithelial cells differentiated into the conjunctival epithelium. When transplanted to the eyes of rabbits with defective conjunctivas, the bioengineered conjunctivas, including PAS-positive goblet cells, were completely grafted, showing good tissue biocompatibility. The transplanted cells survived and maintained an aligned regular morphology. However, this study did not report for how long the transplanted cells remained viable and expressed conjunctival markers. Although this pilot study showed promising results, they were far from demonstrating a truly functional ocular surface reconstruction.

Another more recent example was published by Bertolin et al. in 2019 [83]. They presented a protocol for preparing autologous tissue-engineered conjunctival epithelial sheets free of all animal components. They used AM and fibrin tissue gel as scaffolds to culture human conjunctival cells obtained from different conjunctival areas. Cells biopsied from the inferior forniceal area demonstrated higher percentages of stem cells, resulting in the best area for isolating cells having a high regenerative capacity in terms of the expression of specific markers and growing on the scaffolds. The authors found variability depending on the AM batch. This, along with the difficulties in accomplishing quality control before releasing the graft, is the main hurdle the authors identified in standardizing a medical product composed of conjunctival cells grown on AM. In addition, they also observed holes in the AM while cells were growing, which could affect the integrity of the surface. Regarding the fibrin glue gel, the authors considered it the ideal scaffold, as it is already a transparent pharmaceutical product, and the quality control tests could be performed without affecting the final product, although low numbers of goblet cells were identified and small amounts of MUC5AC were measured. The impact of this paper is limited, as no in vivo experiments for regenerating conjunctival defects were carried out.

Finally, there was a recent study [84] in which a 3D-printed gelatin/elastin/hyaluronic acid membrane was designed for conjunctival reconstruction. The overall aim was to replace the use of AM as a graft in ocular surface reconstruction because of its well-known limitations. An in vivo evaluation was conducted that involved implanting the bioprinted membranes and AM on induced conjunctival defects in rabbits. Although the constructs showed physical and mechanical characteristics adequate for successful ocular surface defect reconstruction, and the authors claimed that their membrane could be considered a promising alternative to AM, this first attempt had an important limitation: it completely lacked the cellular component. The endpoint of the study was only the morphological quality of the healed conjunctiva after the membrane transplant. Although the bioengineered membrane may work as a wound dressing, the functional regeneration of conjunctival tissue would not be achieved.

We are still far from achieving a bioengineered complete human conjunctival tissue replacement, but these promising studies are paving the way towards that goal.

### 3.2. Gene Therapy

Gene therapy has great potential to prevent conjunctival bleb fibrosis associated with the failure of glaucoma filtering surgery. However, there are very few examples of gene therapy developments for treating this problem. We next mention several published papers related to this topic using animal or human-derived materials.

The use of antimetabolites, such as mitomycin-C and 5-fluorouracil, as conjunctival anti-scarring agents in glaucoma filtering surgery began in the 1990s; however, their potentially blinding side-effects, such as wound leakage, hypotony, and infection, along with their indiscriminate effects on cells render conjunctival scarring a not-yet-resolved problem of high clinical relevance.

As TGFβ is the main fibrogenic signal that modulates the fibrotic process [61,62], interfering with the signaling pathway that TGFβ1–β3 use to induce fibrosis could be a good strategy for preventing or treating conjunctival fibrosis. Smad7 gene transfer, a member of the Smad signaling pathway, was reported as a potential strategy with which to modulate the fibrotic reaction that occurs in an incision-injured mouse conjunctiva during the healing process [85,86]. The authors first showed that Smad7 overexpression delivered using an adenoviral vector inhibited the TGFβ1-driven upregulation of both fibrogenic and inflammatory components in cultured human subconjunctival fibroblasts [85]. All this suggests the therapeutic potential of adenovirus-based Smad7 gene transfer to prevent excess scarring from trabeculectomy.

A recent review published by Komáromy et al. [87] clearly summarizes the more advanced developments in this field. There have been several successful examples in experimental models, but few techniques have reached the clinical trial stage in humans. An example is the development of a small interfering RNA to silence transcription factors involved in conjunctival tissue fibrosis, such as the myocardin-related transcription factor/serum response factor (MRTF/SRF) pathway or secreted protein acidic and rich in cysteine (SPARC) [88,89,90]. Another example is the presurgical subconjunctival injection or topical administration onto the surgical field of recombinant adenovirus with the human p21 transgene (encoding the CDKN1A protein) in rabbits [91]. The modulation of wound healing after trabeculectomy would be achieved in this case by the cell cycle arrest of surrounding cells rather than their destruction using conventional mitomycin-C. Finally, the adenovirus-mediated blockage of p38 mitogen-activated protein kinase (MAPK) resulted in the inhibition of the fibrogenic reaction induced by the subconjunctival fibroblasts in mice with conjunctival scarring [86].

In humans, one strategy studied was gene delivery using an anti-sense oligonucleotide that specifically inhibits the gene expression of TGFβ2(ISTH0036) [92], which has shown promising results in open-angle glaucoma patients undergoing trabeculectomy. Patients received a single dose of ISTH0036 at the end of surgery by intravitreal injection. The results of the study showed that ISTH0036 was safe, as there were no adverse events directly related to the ISTH0036 injection. Additionally, single-dose ISTH0036 administration resulted in intraocular pressure values < 10 mmHg that were maintained over the three-month postoperative observation period. This is the first clinical study that shows the clinically relevant results of a gene therapy product that displays a potent anti-fibrotic effect in the conjunctiva. It may be worth exploring its application in other forms of fibrotic diseases in which the conjunctiva is involved.

## 4. Concluding Remarks

The conjunctiva is an essential tissue for maintaining a healthy ocular surface. The great complexity of this tissue, along with the effect of neglecting it in favor of the cornea and limbus, accounts for the delay in the development of ATMPs that target the conjunctiva. Nevertheless, as described in this review, important advances have been made in the last few years, especially in the field of tissue engineering. Further interesting studies in this field are anticipated in the next few years.

## Figures and Tables

**Table 1:** Summary of the main conjunctival diseases that may require tissue transplantation.

Disease	Mechanism that May Necessitate Transplantation
Infectious conjunctivitis: – Adenoviral – Streptococcal – Trachoma	Fibrosis of the upper tarsal conjunctiva can lead to corneal pathology when blinking. Surgical removal of cicatrizing tissue may require conjunctival tissue-like transplantation.
Atopy-related conjunctivitis: – Vernal keratoconjunctivitis – Atopic keratoconjunctivitis	Fibrosis is possible, but not frequent.Surgery to remove giant papillae in VKC, rarely needed, could necessitate conjunctival tissue-like transplantation.
Autoimmune cicatrizing conjunctivitis: – Mucous membrane pemphigoid (ocular cicatricial pemphigoid)	Intense progressive fibrosis leading to fornix shortening and symblephara may necessitate reconstructive surgery and, thus, conjunctival tissue-like transplantation, especially if further limbal stem cell therapy-like and/or corneal transplant is needed.
Immune-based conjunctivitis: – Graft vs. host disease – Rosacea-related – Sjögren-associated DED – Stevens–Johnson syndrome (SJS) and its spectrum	Intense fibrosis leading to symblephara and corneal pathology, most likely in SJS, may necessitate conjunctival tissue-like transplantation after its removal. Mostly required if stem cell transplantation and/or corneal transplant is planned.
Multiple mechanisms involved: – Pterygium	Conjunctival tissue-like transplantation is always required after surgical removal.
Extensive benign and malignant tumors: – Epithelial tumors – Lymphoid hyperplasia/lymphoma – Melanocytic tumors	Conjunctival tissue-like transplantation may be required after surgical removal if extensive areas of the conjunctiva are removed.
Trauma- and surgery-related pathology: – Chemical injury – Multiple glaucoma filtering surgeries – Periorbital reconstruction	If extensive fibrosis makes the removal of tissue necessary, then conjunctival tissue-like transplantation can be considered.
